# The Genome of the Medicinal Macrofungus *Sanghuang* Provides Insights Into the Synthesis of Diverse Secondary Metabolites

**DOI:** 10.3389/fmicb.2019.03035

**Published:** 2020-01-14

**Authors:** Ying Shao, Hongwei Guo, Jianping Zhang, Hui Liu, Kun Wang, Song Zuo, Pengfei Xu, Zhenrong Xia, Qiumei Zhou, Hanghang Zhang, Xiangqing Wang, Anhui Chen, Yulong Wang

**Affiliations:** ^1^Jiangsu Key Laboratory of Food Resource Development and Quality Safe, Xuzhou University of Technology, Xuzhou, China; ^2^Jiangsu Konen Biological Engineering Co., Ltd., Nanjing, China; ^3^Experimental Center of Clinical Research, The First Affiliated Hospital of Anhui University of Chinese Medicine, Hefei, China; ^4^Nanling Forestry Technology Center, Nanling Forestry Bureau, Nanling, China; ^5^Department of Neurology, The People’s Hospital of Pingyi County, Pingyi, China; ^6^Anhui Provincial Key Laboratory of Microbial Pest Control, Anhui Agricultural University, Hefei, China

**Keywords:** *Sanghuang*, genomics, CAZymes, bioactive compound biosynthesis, transporters

## Abstract

The mushroom, *Sanghuang* is widely used in Asian countries. This medicinal fungus produces diverse bioactive compounds and possesses a potent ability to degrade the wood of the mulberry tree. However, the genes, pathways, and mechanisms that are involved in the biosynthesis of the active compounds and wood degradation by *Sanghuang* mushroom are still unknown. Here, we report a 34.5 Mb genome—encoding 11,310 predicted genes—of this mushroom. About 16.88% (1909) of the predicted genes have been successfully classified as EuKaryotic Orthologous Groups, and approximately 27.23% (665) of these genes are involved in metabolism. Additionally, a total of 334 genes encoding CAZymes—and their characteristics—were compared with those of the other fungi. Homologous genes involved in triterpenoid, polysaccharide, and flavonoid biosynthesis were identified, and their expression was examined during four developmental stages, 10 and 20 days old mycelia, 1 year old and 3 years old fruiting bodies. Importantly, the lack of *chalcone isomerase 1* in the flavonoid biosynthesis pathway suggested that different mechanisms were used in this mushroom to synthesize flavonoids than those used in plants. In addition, 343 transporters and 4 velvet family proteins, involved in regulation, uptake, and redistribution of secondary metabolites, were identified. Genomic analysis of this fungus provides insights into its diverse secondary metabolites, which would be beneficial for the investigation of the medical applications of these pharmacological compounds in the future.

## Introduction

*Sanghuang* is a medically important fungi and has been used as a traditional medicine throughout China, Japan, and Korea. The first use of *Sanghuang* was reported in the oldest Chinese medicinal book, Shennong’s Compendium of Materia Medica, which reported the use of *Sanghuang* for the treatment of various disease conditions, such as stomach pain and amenorrhea; the use of this fungus is also correlated with prolonged life, detoxification, and improved digestion after long-term use (Zhu et al., 2008; Lee et al., 2011; Cai et al., 2019). Ikekawa et al. (1968) confirmed that the water extracts of the *Sanghuang* fruiting bodies had potent biological activities and can be used to inhibit sarcoma cell growth in mice (Shibata et al., 1968). Since then, numerous studies focusing on its medicinal functions have been reported, further characterizing the antitumor and antioxidant properties of this fungi (Zhang et al., 2014; Yan et al., 2016). Extensive studies in this regard have shown that the main components of *Sanghuang*, as a medicinal fungus, are considered as effective antioxidant and antitumor drugs (Cai et al., 2019).

Because of its large market demand and the limitation of the slow growth of the fruit, natural *Sanghuang* is in serious short supply; therefore, large scale industrial production of a few *Sanghuang* species is required to eliminate this shortage. However, the fungal name has been controversial for long time owing to the ambiguity of these fungal circumscription with the other fungi species (Wu et al., 2012; Cui, 2014; Zhou et al., 2015). *Sanghuang* has been mistakenly reported as *Inonotus linteus*, *Phellinus igniarius*, and *Phellinus baumii*; Zhou et al. (2015) investigated the phylogenetic relationship of *Inonotus* spp. and defined the three distinct groups as *Inonotus* s.s., *Sanghuangporus*, and *Tropicoporus*. The phylogenetic position, and the morphological and ecological features indicated that the true *Sanghuang* (*I. sanghuang*) is a member of *Sanghuangporus*; thus, the authentic *Sanghuang* should be renamed as *Sanghuangporus sanghuang* (*S. sanghuang*) and characterized as a new species that has been discovered to only grow on the mulberry trees (Wu et al., 2012; Zhou et al., 2015). Thus, it is necessary to clarify the taxonomic position of *Sanghuang* species that are extensively utilized for industrial production based on the molecular phylogenetic analysis.

*Sanghuangporus sanghuang* produces hundreds of different compounds, many of which are bioactive fractions and constituents (Lin et al., 2017; Liu et al., 2017; Cai et al., 2019; Wu et al., 2019). Triterpenoids, polysaccharides, and flavonoids are the three major categories of pharmacologically active compounds in *S. sanghuang*. Polysaccharides, such as water soluble intracellular polysaccharides (IPSWs), homogenous polysaccharides (PIP_1_), and heteropolysaccharides (PIP60-1), have been reported to exert anti-cancer, anti-diabetes, anti-inflammation and antioxidation effects (Jang et al., 2004; Hwang et al., 2005). Triterpenoids play an important role in antitumor, anti-inflammation, and immune regulatory properties (Taji et al., 2008; Wang et al., 2009). Moreover, *S. sanghuang* is one of the rare fungi that contains flavone compounds, which have antioxidation and antitumor effects (Feng et al., 2012; Liu et al., 2017). *S. sanghuang*, as a white rot fungus with wood degradation ability, secretes lots of carbohydrate-active enzymes (CAZymes) for the degradation of the mulberry trees (Zhou et al., 2015).

The availability of fungal genome sequences has facilitated research on gene diversity related to the biosynthesis of the secondary metabolites (Corre and Challis, 2009; Dai, 2019). At present, a large number of medicinal mushroom genomes have been sequenced; these include *Ganoderma lucidum*, *Wolfiporia cocos*, and *Auricularia heimuer* (Chen et al., 2012; Floudas et al., 2012; Dai, 2019). Although *S. sanghuang* has many important properties, but until now only approximately 120 nucleotide sequences available in the National Center for Biotechnology Information (NCBI) database for *S. sanghuang*, and most of them are used for phylogenetic analysis. Therefore, the current genomic sequence resources are not sufficient to reveal the pharmacological mechanisms of *S. sanghuang* at the molecular level.

Here, we report the complete genomic sequence of *S. sanghuang Kangneng*, which has been used for large scale industrial production in China. The genes and gene clusters involved in secondary metabolism and their regulation have been identified in this study. Information regarding the whole genome sequencing of *S. sanghuang* contributes to its medicinal application and future studies.

## Materials and Methods

### Culture Conditions and DNA Isolation

In this study, a total of six *S. sanghuang* strains that have been used for large scale industrial production in China, labeled as *S. sanghuang QianDaoHu*, *S. sanghuang Korea*, *S. sanghuang BeiJing*, *S. sanghuang JinZhai*, *S. sanghuang KangNeng*, and *S. sanghuang ZhangJiaJie*, were obtained from Jiangsu KONEN Biological Engineering Co. Ltd (Anhui, China). The single spore strains were obtained following the methods of Choi et al. (1999). All the strains were cultured using potato dextrose agar (PDA) at 28°C and are available upon request. The single spore mycelia were used for genomic DNA extraction by the cetyltrimethyl ammonium bromide (CTAB) method.

### Phylogenetic Analysis

The internal transcribed spacer (ITS) region was amplified using a primer pair of ITS4 and ITS5, and was sequenced from six strains as reported previously (Tsai et al., 2017). The sequences were aligned using the ClustalW application included in BioEdit^1^. The alignment was converted to MEGA file and a phylogenetic tree was constructed and produced by Maximum likelihood using MEGA X^2^ software of the bootstrap values (1000 replicates).

### Genome Sequencing and Assembly

*Sanghuangporus sanghuang KangNeng* was selected for genome sequencing as it is the most widely cultured strain among the six *S. sanghuang* strains. The DNA extracted from *S. sanghuang KangNeng* was used for genome sequencing. Illumina HiSeq and the PacBio sequencing approach were combined for the whole genome sequencing to obtain better sequencing. Genome assembly was performed as previously reported (Zhang et al., 2018). Briefly, raw reads from PacBio were filtered out, and Proovread 2.14.0 was used for the subread correction (>1 Kb); then, SMRT Analysis v.2.3.0 was used for assembly. The completeness of this assembly was assessed using the Benchmarking Universal Single-Copy Orthologs v3 (BUSCO), as described previously (Seppey et al., 2015).

### Genome Annotations

Tandem Repeats Finder (TRF 4.04) was used to search for tandem repeats in all scaffolds. Transposable element (TE) annotation was performed with RepeatMasker 4.06. The tRNAscan-SE 1.3.1 and RNAmmer 1.2 were used for the identification of the tRNAs and rRNAs, and Genemark-ES 4.21 was used for *de novo* gene predictions (Ter-Hovhannisyan et al., 2008).

### Functional Annotation

The functional annotations of the genes were performed according to databases, including CAZymes (Carbohydrate-Active enZYmes Database), P450, and KOG (Eukaryotic Orthologous Groups) (Tatusov et al., 2003; Cantarel et al., 2009; Magrane and UniProt Consortium, 2011). Fungal Transcription Factor Database was used for transcription factors annotation (Wilson et al., 2008). The antiSMASH analyses^3^ were run with default parameters (relaxed) to predict the secondary metabolite biosynthesis gene clusters in the fungi.

### Quantitative PCR (qPCR)

For the collection of different samples in asexual development, the strain was inoculated onto PDA at 28°C in the dark; all mycelia on the plate were collected and grinded into powder using liquid nitrogen for RNA extraction. For fruiting body production, the mycelia of this strain were inoculated into a 250 ml flask containing 100 ml liquid PDS (20% potato, 2% Mulberry tree sawdust) medium. The flask was then incubated at 28°C in a 200 rpm shaker for 10 days. Sterilized short log sections were inoculated with the fungal culture from the same flask by the top spawning method (Hur, 2008), cultivated in the dark at 30°C with relative humidity of 96% for 2 months, and then maintained at 30°C under a 17:7 h dark/light cycle until collection. Whole fresh fruiting bodies were cut into small pieces and pulverized to powder using a grinder, then further grinded into powder using liquid nitrogen for RNA extraction. Total RNA was extracted at different asexual developmental stages of *S. sanghuang*, i.e., 10 days old (M10d) and 20 days old (M20d), as well as from different sexual developmental stages, i.e., one year old (OY) and three year old (TY). The cDNA was synthesized and quantitative PCR was performed as reported previously (Shao et al., 2019). qPCR amplification was performed with a SYBR Green kit (Takara) and Bio-Rad CFX96TM system (Hercules, CA, United States). The stability of gene expression of actin, β-tubulin, α-tubulin and glyceraldehyde-3-phosphate dehydrogenase (GAPDH) was determined in M10d, M20d, OY, and TY, and both α-tubulin and GAPDH were found to be stable in different fungal stages. Therefore, GAPDH was used as the internal control in this study. The expression values were obtained by normalizing to that of the control gene and the 2^–Δ^ ^Δ^ ^Ct^ method was used for calculating the relative gene expression levels. All the experiments were performed in six replicates, and data are presented as the mean ± SE of six replicates. Primers are listed in Supplementary Table S1.

### Data Availability Statement

The genome assembly of *S. sanghuang Kangneng* has been deposited at NCBI with the accession no. VVIT00000000.

## Results

### Molecular Phylogeny of Six Selected Strains

**FIGURE 1:**
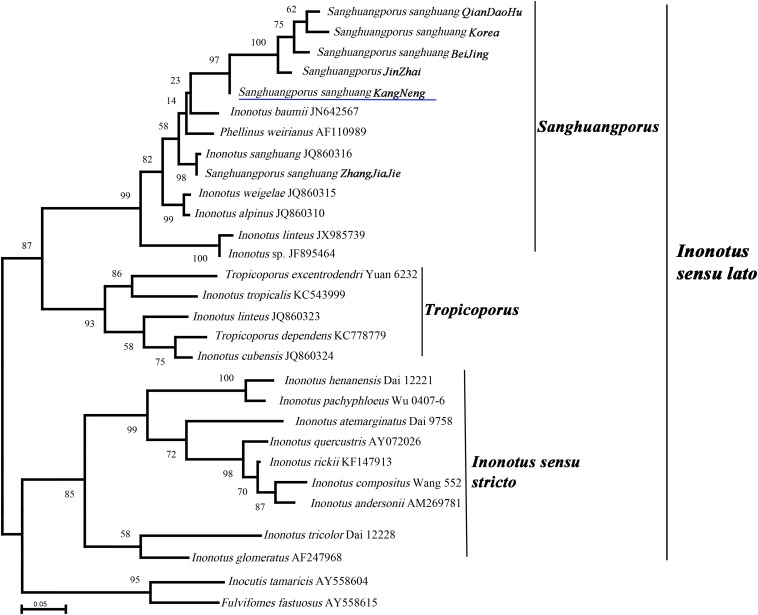
Phylogeny of *Sanghuangporus sanghuang* strains and selected representative fungi inferred from ITS sequences. Bootstrap values from maximum likelihood analysis. The newly generated sequences for this study have been submitted to GenBank with accession numbers (MN242716–MN242721). The underlined species name was genome-sequenced in this study.

*Sanghuang* has long been misleadingly referred to by some incorrect binomial names, and the species concept of *sanghuang* has only recently been established by molecular phylogenetic studies; thus, it is necessary to clarify the taxonomic position of *sanghuang* strains that are extensively utilized in China. Therefore, six *S. sanghuang* strains that are used for large scale industrial production were selected, and the phylogenetic analysis of their ITS nrDNA sequences was performed. In the phylogenetic analysis (Figure 1), species of *Inonotus* formed at least three clades (*Sanghuangporus*, *Tropicoporus*, and *Inonotus sensu stricto*), and all the six selected strains were included in the *Sanghuangporus* clade, suggesting that these commercially available *sanghuang* mushrooms in China are *S. sanghuang* as opposed to others (Figure 1). Further analysis showed that the five *S. sanghuang* species (*S. sanghuang QianDaoHu*, *S. sanghuang Korea*, *S. sanghuang BeiJing*, *S. sanghuang JinZhai*, and *S. sanghuang KangNeng*) are more closely related to each other than *S. sanghuang Zhangjiajie*, revealing that these commercially available *sanghuang* mushrooms in China are diverse.

### Genome Sequencing, Assembly, and Annotation

**FIGURE 2:**
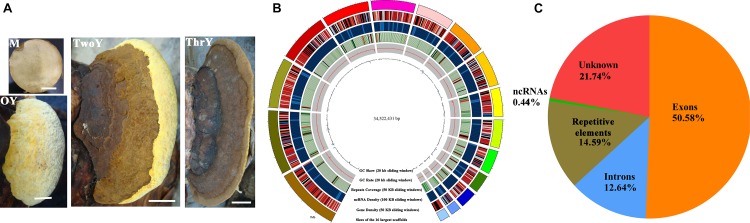
Growth characteristics and general genomic features of *S. sanghuang Kangneng*. **(A)** The four developmental stages in the life cycle of the fungus. M, aerial mycelia; OY, one-year-old fruiting bodies; TwoY, two-year-old fruiting bodies; ThrY, three-year-old fruiting bodies. Bar = 3 cm. **(B)** Characteristics of the *de novo* assembly genomic features. From the outside to the inside are: I, scaffolds, the different colors represent different scaffolds (scaffolds length > 1 Mbp were chosen); II, gene density; III, the density of non-coding RNA; IV, percentage of coverage of repetitive sequences; V, GC content estimated by the percentage of G + C in 20 kb; VI, GC skew was estimated with (G–C)/(G + C) in 20 kb. **(C)** Genomic element density.

*Sanghuangporus sanghuang KangNeng* was selected for genome sequencing as it is the most widely cultured among the six *S. sanghuang* strains. First, the characteristics at four different developmental stages (mycelia and fruiting bodies of 1, 2, and 3 year old) were evaluated; the fungi were perennial basidiocarps, effused-reflexed to pileate, and had a brown, dark grayish to black, pileal surface, which is in accordance with the characteristics previously reported (Figure 2A) (Zhou et al., 2015).

A 34.5 Mb genome sequence was obtained (∼440 X coverage), and the assembly consisted of 37 scaffolds with 47.95% GC content (Table 1 and Figure 2B). In total, 11,310 genes were predicted, with an average sequence length of 1,930 bp, and accounted for 63.22% of the whole genome sequence length, revealing a high gene density in the genome (Table 1 and Figure 2A). There were 66,421 predicted exons, and the cumulative length accounted for 50.58% of the whole genome sequence length, and 55,111 introns were predicted. There were 251 non-coding RNAs (ncRNAs), such as tRNA, rRNA, and small RNA that were found; these ncRNAs represented 0.45% of the whole genome length, suggesting that ncRNA formed only a small proportion of the overall genome size (Table 1 and Figure 2C).

**TABLE 1:** Genome assembly and features of *S. sanghuang Kangneng.*

**Scaffold characteristics**	
Total number	37
Total length (bp)	34,522,431
N50 Length (bp)	2,512,777
N90 Length (bp)	579,196
Coverage	440
Max length (bp)	3,684,693
Min length (bp)	21,097
% G + C content	47.95
**Genome characteristics**	
Genome assembly (Mb)	34.5
Protein-coding genes	11,310
Number of exons	66,421
Number of introns	55,111
Average length of coding sequence genes (bp)	1929
Average exon length (bp)	263
Average intron length (bp)	79
Average number of exons per genes	5.87
Repeat Size (bp)	5,037,389
TE Size (bp)	4,943,475
tRNA	67

The total length of the repeat sequences was 5,037,389 bp, covering 14.59% of the genomic length. Tandem repeat sequences only accounted for 0.27%, and transposable elements (TEs) accounted for approximately 14.32% of the genome. Among the TEs, long terminal repeats (LTRs) and non-LTR transposons accounted for 9.84 and 4.48% of the genome, respectively (Table 1).

### Gene Function Annotation

**FIGURE 3:**
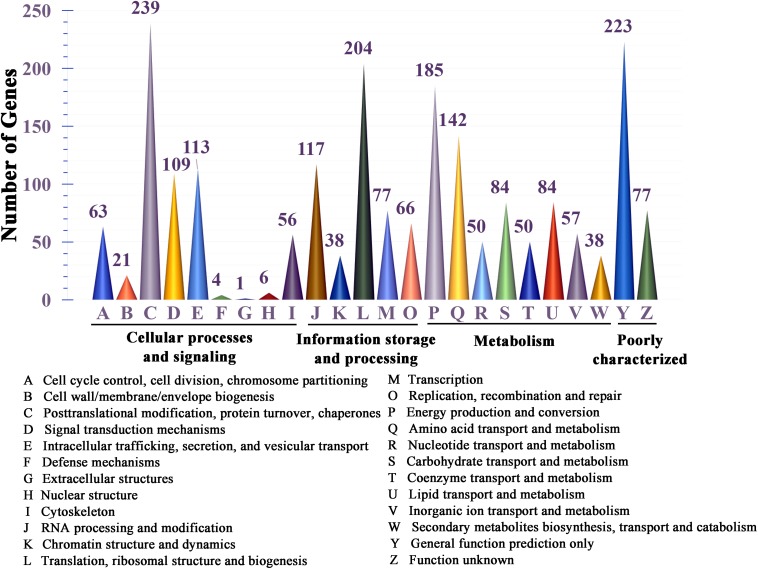
KOG functional classification of proteins in the *S. sanghuang Kangneng* genome.

All 11,310 protein-encoding genes were annotated in this study (Supplementary Table S1). Among all of the predicted genes, 2,442 genes were annotated by the KEGG pathway, out of which 43.28% (1057) were involved in metabolism, accounting for the major proportion (Supplementary Table S2). Genes classified into functional categories based on COG analysis accounted for 9.45% (1069), and within in these genes, 59.31% (634) of the total predicted genes were involved in metabolism (Supplementary Table S3). A total of 16.88% (1909) of the genes could be classified in the KOG analysis; the results showed that 27.23% (665) of these genes are involved in metabolism, and genes involved in “energy production and conversion,” “amino acid transport and metabolism,” “carbohydrate transport and metabolism” and “lipid transport and metabolism” accounted for the major proportion among these metabolism-associated genes (Figure 3 and Supplementary Table S4).

### Functional Annotation of Putative CAZymes

As a white rot fungus with wood degradation ability, CAZymes were identified in the *S. sanghuang* genome and were compared with the other fungi. A total of 334 genes could be assigned to CAZyme families as defined in the CAZy database (Supplementary Table S5). All CAZymes were classified into 5 major modules; 185 genes for glycoside hydrolases (GH), 69 genes for glycosyl transferases (GT), 12 genes for polysaccharide lyases (PL), 61 genes for carbohydrate esterases (CE), and 7 genes for carbohydrate-binding modules (CBM) (Supplementary Table S5).

**FIGURE 4:**
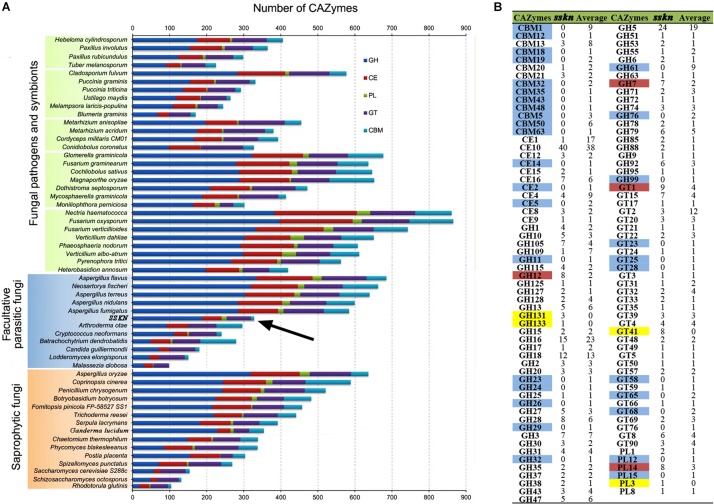
Comparative analysis of CAZymes from *S. sanghuang Kangneng* and other fungi. **(A)** Comparative analysis of fungal CAZymes. The numbers of CAZyme modules or domains are represented as horizontal bars. **(B)** Average number of CAZymes from a total of 30 Basidiomycota fungi. Blue, CAZyme present in other Basidiomycota fungi but not in *SSKN*; Red, number of CAZymes present in *SSKN* greater than one-fold the average number of other Basidiomycota fungi; Yellow, CAZymes only present in *SSKN*. *SSKN*, *S. sanghuang Kangneng*.

To study the characteristics of CAZymes in *S. sanghuang*, CAZymes from fungi *Ascomycota*, *Basidiomycota*, *Chytridiomycota*, and *Zygomycota* were analyzed together, as previously reported (Figure 4A) (Zhao et al., 2013). In the *S. sanghuang* genome, the number of CAZymes is in the middle among all of the selected fungi but is different from most other fungi, and the number of GHs was remarkably larger than the number of GTs, suggesting that fungal survival is dependent on lignocellulose decomposition. It was concluded that polysaccharide decomposition is more important than polysaccharide construction for the growth and metabolism of *S. sanghuang* (Figure 4A).

Moreover, CAZymes from a total of 30 *Basidiomycota* were identified and compared with those of *S. sanghuang* (Figure 4B and Supplementary Table S6). Although the results showed that the gene numbers in the 5 major modules of CAZymes were similar in *Basidiomycota* fungi, GH131, GH133, GT41, and PL3 were specifically present in *S. sanghuang*, and the copy numbers of GH12, GH7, GT1, and PL14 in the fungi were remarkably higher than those in other *Basidiomycota* fungi, suggesting that these genes may be involved in mulberry tree cell wall-degradation (Figure 4B and Supplementary Table S6).

### The Biosynthesis of Bioactive Compounds in *S. sanghuang*

*Sanghuangporus sanghuang* produces diverse secondary bioactive metabolites, such as triterpenoids, polysaccharides, and flavonoids, which are its main components that have immunomodulatory, antioxidant, and antitumor activities (Lin et al., 2017; Liu et al., 2017). Therefore, genes involved in the biosynthesis of these secondary metabolites were searched in the fungal genome.

#### Triterpenoid Biosynthesis

**FIGURE 5:**
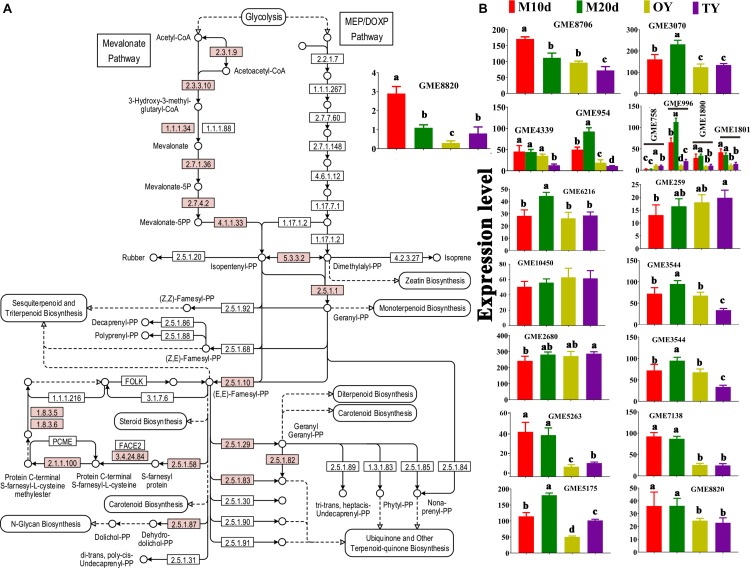
KEGG mapping of the terpenoid backbone biosynthesis pathway and differential expression of the enzymes identified in *S. sanghuang Kangneng*. **(A)** The red box indicates existing homologous genes of the enzyme. **(B)** The expression levels of genes in different developmental stages. Different lowercase letters marked on the bars in each graph denote significant differences (*P* < 0.05).

A total of 18 key enzymes involved in the mevalonate pathway (MVA) pathway were identified in this study (Figure 5A and Table 2). The hydroxymethylglutaryl-CoA (HMG-CoA) synthase, geranyl-diphosphate synthase, diphosphate synthase, and terpenoid cyclase enzymes are each encoded by two or more copies of the respective genes, whereas the other 13 enzymes are encoded by single copy genes (Table 2). A gene (GME6608) encoding lanosterol synthase (*LSS*) was found, and it catalyzes the cyclization of the triterpenes squalene or 2-3-oxidosqualene to a protosterol cation and finally to lanosterol, the precursor of all steroids (Dai, 2019).

**TABLE 2:** Terpenoid backbone biosynthesis (Total = 18).

**Enzyme**	**Ezyme ID**	**ID**
acetyl-CoA acyltransferase	ec:2.3.1.9	GME8706
HMG-CoA synthase	ec:2.3.3.10	GME4339, GME954
reductase (NADPH)	ec:1.1.1.34	GME6216
Kinase	ec:2.7.1.36	GME10450
Prenylcysteine lyase	ec:1.8.3.5	GME2680
Prenylcysteine lyase	ec:1.8.3.6	GME2680
mevalonate phosphate kinase	ec:2.7.4.2	GME5263
Decarboxylase	ec:4.1.1.33	GME5175
delta-isomerase	ec:5.3.3.2	GME3070
geranyl-diphosphate synthase	ec:2.5.1.1	GME758, GME996, GME1800, GME1801
terpenoid cyclases	ec:2.5.1.58	GME9472, GME7025
hexaprenyl-diphosphate synthase	ec:2.5.1.82	GME259
hexaprenyl-diphosphate synthase	ec:2.5.1.83	GME259
polycis-polyprenyl diphosphate synthase	ec:2.5.1.87	GME3544
diphosphate synthase	ec:2.5.1.10	GME758, GME996, GME1800, GME1801
diphosphate synthase	ec:2.5.1.29	GME1800, GME1801
STE24 endopeptidase	ec:3.4.24.84	GME7138
O-methyltransferase	ec:2.1.1.100	GME8820

The expression of the genes in the terpenoid backbone synthesis pathway was detected with qPCR to predict the triterpenoid production at different stages of the fungus (Figure 5B). The results showed that most of these genes were differentially expressed between the mycelium and fruiting body, and most of the genes, *LSS* included, were expressed at the highest levels in the mycelial stages (M10d or M20d), suggesting that the fungi may produce more triterpenoids in the mycelial stages than in the fruiting body stages (Figure 5B).

#### Polysaccharide Biosynthesis

**FIGURE 6:**
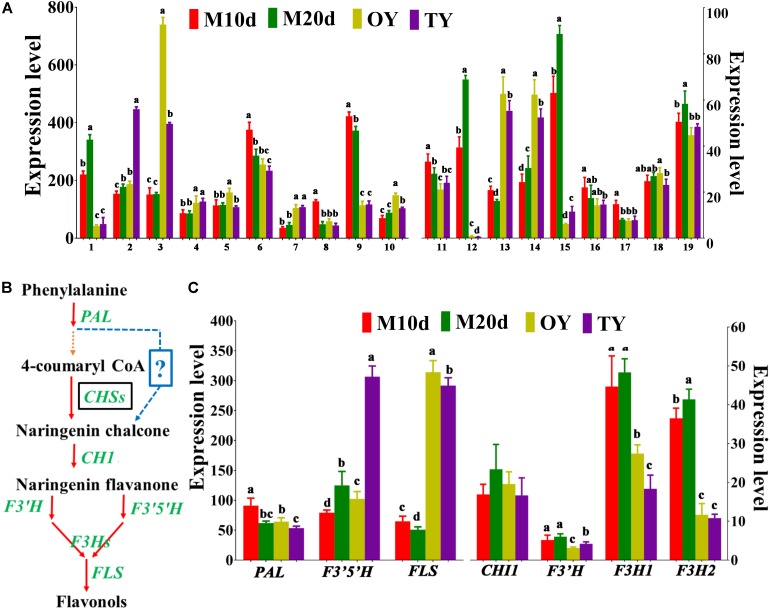
Differential expression of the enzymes involved in polysaccharide and flavonoid biosynthesis identified in *S. sanghuang Kangneng*. **(A)** The expression levels of genes involved in polysaccharide biosynthesis in different developmental stages. **(B)** Putative pathway of flavonoid biosynthesis in *S. sanghuang Kangneng*. Black box, no homologous gene was found in the fungal genome; ?, unknown reactions in the fungus. **(C)** The expression levels of genes involved in flavonoid biosynthesis in different developmental stages. Different lowercase letters marked on the bars in each graph denote significant differences (*P* < 0.05).

Polysaccharides that account for the pharmaceutical potential of *S. sanghuang* are another kind of highly bioactive compounds. Among all the polysaccharides in the medicinal fungi, the water-soluble 1,3-β-and 1,6-β-glucans are the most active as immunomodulatory and antioxidant compounds (Chen et al., 2012; Mizuno and Nishitani, 2013). The two genes encoding 1,3-β-glucan synthases and seven genes encoding β-glucan biosynthesis-associated proteins were identified in the genome of *S. sanghuang*; all these proteins contain a SKN1 domain that plays important roles in 1,6-β-glucan biosynthesis, suggesting the same mechanism in fungal polysaccharide biosynthesis (Supplementary Table S7) (Shahinian and Bussey, 2000; Douglas, 2001; Chen et al., 2012). A total of 19 genes involved in polysaccharide biosynthesis and their regulation were found based on the fungal genome; the qPCR results showed that although all of these genes were differentially expressed between the mycelium and fruiting body, approximately half of these genes (10/19) were expressed at the highest levels in the mycelial stages (M10d or M20d), while the others were expressed at their lowest levels during this stage, revealing that further studies should be performed to examine the differences in polysaccharide production at diverse stages of the fungus (Figure 6A).

#### Flavonoid Biosynthesis

*Sanghuangporus sanghuang* is considered as one of the few species of the fungi that is capable of yielding flavonoids, and can produce various flavonoid compounds that have antioxidant, antiproliferative, and antimicrobial activities (Liu et al., 2017). Putative genes involved in flavonoid biosynthesis were searched based on a previous study, and 7 core genes involved in flavonoid biosynthesis were found (Supplementary Table S8) (Saito et al., 2013). In plants, chalcone synthase (CHS) is the first enzyme specific for the flavonoid pathway; however, no gene encoding CHS was found in the *S. sanghuang* genome, suggesting a different catalytic mechanism for naringenin chalcone production in this fungus (Figure 6B) (Falcone Ferreyra et al., 2012). The other key genes, *chalcone isomerase 1* (*CHI1*), *flavonoid-3′, 5-hydroxylase* (*F3′5′H*), *F3′H -flavonoid-3′-hydroxylase* (*F3′H*), *flavanone-3-hydroxylase 1* (*F3H1*), *flavanone-3-hydroxylase 2* (*F3H2*), and *flavonol synthase* (*FLS*), were present in the fungus, revealing that the central pathway for flavonoid biosynthesis is conserved between this fungus and the plants (Figure 6B and Supplementary Table S9).

The expression levels of genes involved in flavonoid biosynthesis (*PAL*, *CHI1*, *F3′H*, *F3′5′H*, *F3H1*, *F3H2*, and *FLS*) were examined, and the results showed that six genes (except *F3′H*) were differentially expressed at different fungal development stages, four genes (*F3′5′H*, *F3H1*, *F3H2*, and *FLS*) that are downstream of the pathway were expressed most highly in the mycelial stages (M10d or M20d), than the others, revealing that the fungi may produce more flavonoids in the mycelial stages than in the fruiting body stages (Figure 6C).

### Prediction of Gene Clusters Involved in Bioactive Secondary Metabolite Biosynthesis of *S. sanghuang*

In fungi, genes involved in secondary metabolite biosynthesis are always organized in discrete clusters, thus secondary metabolite biosynthesis gene clusters in the fungus were identified using the antiSMASH software (Supplementary Table S10). A total of 21 gene clusters located in different scaffolds were detected. One non-ribosomal peptide synthase (NRPS) and three NRPS-like gene clusters were identified in the *S. sanghuang* genome, and further analysis showed that the fungal genome encodes 14 terpene synthase (TS), 5 NRPS, and 43 polyketide synthase (PKS) genes (Supplementary Table S11). These data suggested that unlike the medicinal fungi *G. lucidum* and *A. heimuer*, which include a few putative NRPS and PKS genes but may not produce NRP and polyketides, NRPS and PKS are numerous and necessary for *S. sanghuang* (Chen et al., 2012; Dai, 2019). The terpene synthase family is a mid-sized family working on the biosynthesis of monoterpene, sesquiterpene, and diterpene backbones (Chen et al., 2011). A total of 14 TS genes and 14 TS gene clusters were identified in the fungal genome, suggesting that diverse secondary metabolites can be produced in the fungus (Supplementary Tables S10, S11).

### Regulation of Secondary Metabolism in *S. sanghuang*

Velvet family proteins could regulate fungal secondary metabolite production in response to different environmental conditions. In this study, four genes encoding velvet domain containing proteins (VelB, VelA, VosA, and VelC) and the methyltransferase domain containing protein (LaeA) were also identified in the *S. sanghuang* genome (Supplementary Table S12). VelA and VelB interact with LaeA and regulate the secondary metabolism and development in other fungi, suggesting that the pathway for secondary metabolism and development is conserved in fungi (Bayram et al., 2008).

### Transporters in the *S. sanghuang* Genome

In total, 368 transport proteins from 98 families were found in *S. sanghuang*, including 51 ATP dependent transporters, 18 ion channels, and 299 secondary transporters (Supplementary Table S13). It has been reported that MFS transporters play important roles in secondary metabolism, and the ATP-binding cassette (ABC) contribute to polysaccharide transportation (Platta and Erdmann, 2007). In the *S. sanghuang* genome, a total of 299 secondary transporters were found, which are the most abundant among these transporters, 43 of them belonging to the MFS family, indicating MFS family proteins play roles in the secondary metabolite biosynthesis.

## Discussion

Limited genetic information on *S. sanghuang* has hampered its pharmacological applications, although the fungus has antioxidation and antitumor effects (Feng et al., 2012; Liu et al., 2017). Thus, genome sequencing and gene annotation are a critical tool for the research community. Here, we present the genome sequence of *S. sanghuang Kangneng* generated by Illumina and the PacBio RSII long-read sequencing technologies. Because the species concept of *sanghuang* has long been misleading, six of the *S. sanghuang* strains used for large scale industrial production were subjected to phylogenetic analysis, which confirmed that these six strains are the true sanghuang, according to recent molecular phylogenetic studies (Zhou et al., 2015; Han et al., 2016). *S. sanghuang Kangneng* was selected for genome sequencing as it is the most widely cultured among these six strains in China.

The total assembly size was 34.5 Mb, which was lower than that of the other white rot fungi, such as *G. lucidum* and *Lentinus tigrinus* (Chen et al., 2012; Wu et al., 2018). The assembly genome consisted of 37 scaffolds with a 47.95% GC content, which is similar to *Penicillium subrubescens* but contrary to that of the most fungi with a >50% GC content (Peng et al., 2017; Wu et al., 2018). In total, 11,310 gene models were predicted, which accounted for 63.22% of the whole genome sequence length, while only ∼21.74% formed the “Unknown” regions, including promoter, terminator, and other regulatory regions of the genes, suggesting high gene density and low intergenic regions in the genome. The majority of the repetitive content in the *S. sanghuang* genome was made up of TEs (14.32%), and among these TEs, the proportion of long terminal repeats (LTRs) was 9.84%, suggesting LTRs may play critical roles in fungal asexual and sexual development.

Main bioactive components identified from *S. sanghuang* are polysaccharides, flavonoids, and triterpenoids (Cai et al., 2019). Genes involved in polysaccharide, flavonoid, and triterpenoid biosynthesis were identified in this study. As other eukaryotes, *S. sanghuang* triterpenoids are synthesized by the MVA pathway (Chen et al., 2012). For polysaccharide biosynthesis, 1,3-β-glucan synthases and seven β-glucan biosynthesis associated proteins were found in the fungus; these genes play key roles in the biosynthesis of 1,6-β-glucans and are well conserved in different fungi (Shahinian and Bussey, 2000; Douglas, 2001; Chen et al., 2012). *S. sanghuang* was considered to be capable of yielding flavonoids; 7 putative core genes for flavonoid biosynthesis were found based on a previous study of the plants because there have been few reports on fungal flavonoid biosynthesis pathways. CHS enzyme was not found in the *S. sanghuang* genome, suggesting that there is a different catalytic mechanism for naringenin chalcone production in the fungus, which needs to be further elucidated. Moreover, the expression of the genes involved in the biosynthesis of these main components was detected at different stages of the fungus (mycelium and fruiting body), and the results show that the fungi may produce more flavonoids and polysaccharides in the mycelial stages than in the fruiting body stages. Furthermore, some NRPs, PKs, and terpenes biosynthesis associated genes were found in this paper, suggesting that these compounds exist in *S. sanghuang* and their synthesis might be tightly regulated.

## Conclusion

We have sequenced and analyzed the genome of the medicinal macrofungus, *S. sanghuang Kangneng*, which has been widely cultured in China. The exposition of the *S. sanghuang* genome has allowed us to study the biosynthesis of the pharmacologically active compounds produced by these medicinal fungi. Research on the secondary metabolite biosynthesis genes in the *S. sanghuang* genome will accelerate the industrial application of the bioactive molecules. Therefore, information on the *S. sanghuang* genome could contribute to its medicinal applications.

## Data Availability Statement

The datasets generated for this study can be found in the genome assembly of *Sanghuangporus sanghuang Kangneng* has been deposited at NCBI with accession no. PRJNA564179.

## Author Contributions

YW and AC conceived and designed the study. YS, YW, AC, HG, and JZ wrote the manuscript. YS, HG, JZ, HL, KW, SZ, PX, QZ, HZ, ZX, and XW performed the experiments. QZ, XW, and HZ analyzed the data.

## Conflict of Interest

KW, SZ, PX, and ZX were employed by Jiangsu Konen Biological Engineering Co., Ltd. The remaining authors declare that the research was conducted in the absence of any commercial or financial relationships that could be construed as a potential conflict of interest.

## Footnotes

1 http://www.mbio.ncsu.edu/bioedit/bioedit.html

2 https://www.megasoftware.net/download_form

3 https://fungismash.secondarymetabolites.org/#!/start
